# Dietary Cysteamine Supplementation Remarkably Increased Feed Efficiency and Shifted Rumen Fermentation toward Glucogenic Propionate Production via Enrichment of *Prevotella* in Feedlot Lambs

**DOI:** 10.3390/microorganisms10061105

**Published:** 2022-05-26

**Authors:** Qi-Chao Wu, Wei-Kang Wang, Fan Zhang, Wen-Juan Li, Yan-Lu Wang, Liang-Kang Lv, Hong-Jian Yang

**Affiliations:** State Key Laboratory of Animal Nutrition, College of Animal Science and Technology, China Agricultural University, Beijing 100193, China; wuqichao@cau.edu.cn (Q.-C.W.); 18292092306@163.com (W.-K.W.); zhang201605099@163.com (F.Z.); liwjuan1226@163.com (W.-J.L.); wang_yanlu@cau.edu.cn (Y.-L.W.); liangkanglvling@163.com (L.-K.L.)

**Keywords:** cysteamine, feedlot lamb, rumen microorganism, blood metabolites, fermentation

## Abstract

Cysteamine (CS) is an essential nutritional regulator that improves the productive performance of animals by regulating somatotropic hormone secretion. To investigate the fattening potential and effects of CS on rumen microbial fermentation, 48 feedlot lambs were randomly assigned to four groups and fed diets supplemented with different CS concentrations (0, 20, 40, and 60 mg/kg BW). An increase in dietary CS concentrations linearly increased the average daily gain (ADG) and dry matter intake (*p* < 0.05) but decreased the feed-to-gain ratio (*p* < 0.01). For the serum hormone, increasing the dietary CS concentration linearly decreased somatostatin and leptin concentration (*p* < 0.01) but linearly increased the concentration of growth hormone and insulin-like growth factor 1 (*p* < 0.01). Regarding rumen fermentation, ruminal pH, ammonia-N, and butyrate content did not differ among the four treatments, although dietary CS supplementation linearly increased microbial protein and propionate and decreased the amount of acetate (*p* < 0.05). Furthermore, an increase in dietary CS concentrations quadratically decreased the estimated methane production and methane production per kg ADG (*p* < 0.05). High-throughput sequencing revealed that increased dietary CS concentrations quadratically increased *Prevotella* (*p* < 0.05), and *Prevotella* and *norank_f__norank_o__Clostridia_UCG-014* were positively correlated with growth performance and rumen fermentation in a Spearman correlation analysis (r > 0.55, *p* < 0.05). Overall, a CS concentration higher than 20 mg/kg BW produced growth-promoting effects by inhibiting somatostatin concentrations and shifting the rumen toward glucogenic propionate fermentation by enriching *Prevotella*. In addition, *Prevotella* and *norank_f__norank_o__Clostridia_UCG-014* were positively correlated with growth performance in lambs.

## 1. Introduction

As a growth-promoting peptide hormone, growth hormone (GH) is present in a wide range of animal tissues and organs [1]. Its release is regulated in a reciprocal manner by the growth hormone-releasing hormone (GHRH) of the hypophyseal portal system [2]. In ruminants, GH stimulates lipid hydrolysis [3] and induces peripheral blood insulin resistance [4], which leads to the effective retention of more glucose to supply to the host. However, the application of GH for promoting growth in animal husbandry is highly controversial and restricted in many countries because the metabolic residues of this hormone can negatively affect human consumers [5]. The direct addition of exogenous hormones has been rejected, but the indirect improvement of GH concentrations through some regulatory pathways has become a research hotspot. Acting as a growth hormone inhibitor, it reduced somatostatin concentrations in organisms and promoted growth performance by allowing the release of more GH [6]. How to regulate GH indirectly by regulating somatostatin is thus a topic of significant concern.

Cysteamine (CS; β-Mercaptoethylamine), the decarboxylation product of cysteine, is a hormone regulator. CS suppresses somatostatin release [7] and promotes growth by reducing the concentration of somatostatin to regulate the release of GH [8,9]. It also has a significant impact on the antioxidant capacity of animals [10]. Dunshea et al. [11] noted that the dietary supplementation of 6 mg CS per kg of body weight increased dry matter intake (DMI) by 2.76% and daily body weight gain by 7.4% in pigs. In a subsequent study, Barnett and Hegarty [12] noted a higher daily body weight gain and wool production in Merino × Dorset wether lambs that orally received 118 ± 9 g CS daily or 113 ± 11 g/day CS every 3 days, resulting in an improvement in the feed conversion ratio. In another study, dietary CS supplementation at 30 and 45 g/day increased the milk yield and milk protein content in lactating cows [13].

However, whether dietary CS supplementation can alter rumen fermentation and microbiota remains unknown. It was hypothesized that there might be an optimal additional level of CS that could maximize the feed efficiency and improve rumen microbial fermentation. To this end, an experiment involving feedlot lambs was conducted to examine the effects of dietary CS supplementation at different concentrations on growth performance, rumen fermentation, and the bacterial community.

## 2. Materials and Methods

All procedures with animals in the present study were approved by the Animal Ethics Committee of China Agricultural University. The sampling procedures followed the Guidelines on the Ethical Treatment of Experimental Animals (2006) No. 398, established by the Ministry of Science and Technology, China.

### 2.1. Preparation of CS Product

The CS product was purchased from Zhejiang Chengyuan Biotechnology Co., Ltd. (Hangzhou, China), and contained 50% cysteamine, 900 g/kg of dry matter, less than 2 mg/kg of Pb, and less than 2 mg/kg of As. The product was directly coated with corn starch and dextrin to suppress the odor and prevent odor release and stored at 4 °C in a refrigerator prior to the experiment.

### 2.2. Animals, Diet Preparation, and Experiment Design

A total of 48 Dorper × Hu hybrid male lambs at five months of age with an initial live body weight of 33.32 ± 0.94 kg were housed and randomly assigned to four dietary CS treatment groups: CS0, 0 mg/kg BW; CS20, 20 mg/kg BW; CS40, 40 mg/kg BW; or CS60, 60 mg/kg BW. For each treatment group, four pens (2 m × 2 m) with slotted bamboo floors were set up, and three lambs were housed per pen. Fresh water and feed were provided ad libitum. The corresponding amount of CS for each treatment was mixed with the concentrate. All animals received the same basal total mixed rations (TMRs) (Table 1), which were formulated to meet the nutritional requirements of 250 g of daily gain, according to the National Research Council [14]. Foxtail millet silage and corn stover were used as forage. The foxtail millet hybrid (Zhangzagu 2) was locally seeded in the last week of 27 June 2021 and harvested at the heading stage (plant height, 120 cm; harvest height, 10 cm) on 22 September 2021. Immediately, the harvested foxtail millet was chopped into 5 cm portions, mixed with 5% molasses, and baled into foxtail millet silage (diameter, 55 cm; height, 60 cm) with seven layers of bale film via manipulation by an automatic baling and wrapping machine (CB-5552, Shandong, China). The corn stover was obtained from the local grass market (length, 4–5 cm). Corn meal, soybean meal, and rapeseed meal were purchased from the local feed market. Premix was purchased from Beijing Kegao Dabeinong Feed Co., Ltd. (Beijing, China). The feed ingredients were stored with good ventilation for the subsequent experiment. The forage to concentrate ratio was fixed at 20:80. The entire experiment lasted 64 days. All feeds were mixed thoroughly and supplied in a feeder twice daily (08:00 and 17:00) after cleaning to remove contaminated feed (fecal or moisture contamination).

The initial weight of the lambs was recorded at the start of the experiment and then every 21 days before the morning feeding throughout the experimental period. All lambs were allowed to acclimatize to the diet and housing environment prior to the experiment. The actual amount of CS in each diet was readjusted according to the weight data updated every 21 days. Simultaneously, the feed consumption of the lambs in each pen was recorded daily based on the offered and refused feed to calculate the DMI with consideration of the moisture content. The average daily gain (ADG) was calculated as the difference between the initial and final body weight divided by the feeding days, and the feed-to-gain ratio (F:G) was calculated as the DMI divided by the ADG.

### 2.3. Sample Collection

At the end of the experiment, rumen fluid samples were collected from each lamb after 2 h of morning feeding. An oral stomach tube (1.2 m long, 6.0 mm) with negative pressure was used in the process of collecting [15,16] by three skilled experimenters to avoid inducing stress in the animals by pain or mishandling. The samples were then filtered with four layers of cheesecloth and stored at −80 °C for later analysis. Meanwhile, ruminal pH was determined using a portable pH meter. According to the method described by Chaney and Marbach [17], ammonia-N (NH_3_-N) concentrations were determined using a microplate reader (RT-6500, Rayto, Shanghai, China). The concentrations of microbial protein (MCP) were measured based on Bradford and Williams’ method [18]. Metaphosphoric acid (300 μL, 25%, *w*/*v*) was added to 1 mL of the ruminal fluid samples, and these were placed at 4 °C for 30 min. After centrifugation at 10,000× g for 10 min, the supernatant samples were injected into gas chromatography (GC522, Wufeng, Shanghai, China) to measure the volatile fatty acid (VFA).

After collecting the ruminant fluid, blood samples were obtained from the jugular vein using a sterile transfusion needle and an anticoagulated negative pressure blood draw tube containing sodium heparin, and the serum was stored in dry ice after centrifugation (1500× *g*, 12 min). The somatostatin, GH, insulin-like growth factor-1 (IGF-1), and leptin concentrations were determined according to the manufacturer’s instructions for enzyme-linked immunosorbent assay (ELISA) kits (KL-GH-S, Conlon Biotechnology Co., Shanghai, China).

### 2.4. DNA Extraction and Sequencing

Total DNA was extracted from the rumen fluid samples according to the instructions of an E.Z.N.A.^®^ soil DNA kit (Omega Bio-Tek, Norcross, GA, USA). The quality of DNA extraction was determined using 1% agarose gel electrophoresis, and the DNA concentration and purity were determined using a NanoDrop2000. The hypervariable region V3-V4 of the bacterial 16S rRNA gene was amplified with 338F (5′-ACTCCTACGGGAGGCAGCAG-3′) and 806R (5′GGACTACHVGGGTWTCTAAT-3′) by an ABI GeneAmp^®^ 9700 PCR thermocycler (ABI, Los Angeles, CA, USA). The amplification procedure was as follows: initial denaturation at 95 °C for 3 min, followed by 27 cycles of denaturing at 95 °C for 30 s, annealing at 55 °C for 30 s, and extension at 72 °C for 45 s, with a single extension at 72 °C for 10 min, and end at 4 °C. The PCR mixtures contained 5× TransStart FastPfu buffer 4 μL, 2.5 mM dNTPs 2 μL, forward primer (5 μM) 0.8 μL, reverse primer (5 μM) 0.8 μL, TransStart FastPfu DNA Polymerase 0.4 μL, template DNA 10 ng, and finally, ddH_2_O up to 20 μL. PCR reactions were performed in triplicate. The PCR product was extracted from 2% agarose gel and purified using the AxyPrep DNA Gel Extraction Kit (Axygen Biosciences, Union City, CA, USA) according to the manufacturer’s instructions and quantified using a Quantus™ Fluorometer (Promega, Madison, WI, USA).

Purified amplicons were pooled in equimolar and paired-end sequenced on an Illumina MiSeq PE300 platform/NovaSeq PE250 platform (Illumina, San Diego, CA, USA) according to the standard protocols of Majorbio Bio-Pharm Technology Co., Ltd. (Shanghai, China). The raw reads were deposited into the NCBI Sequence Read Archive (SRA) database (Accession Number: PRJNA795301).

The raw 16S rRNA gene sequencing reads were demultiplexed, quality-filtered by FAST version 0.20.0, and merged by FLASH version 1.2.7 [19]. Operational taxonomic units (OTUs) with a 97% similarity cutoff [20,21] were clustered using UPARSE version 7.1 [20], and chimeric sequences were identified and removed. The taxonomy of each OTU representative sequence was analyzed using the RDP Classifier version 2.2 [22] against the 16S rRNA database (e.g., Silva v138) using a confidence threshold of 0.7.

### 2.5. Chemical Analyses

The dry matter (DM), ash, crude protein (CP), and ether extract (EE) of the diet samples were analyzed according to the Association of Official Analytical Chemists [23]. The neutral detergent fiber (NDF) and acid detergent fiber (ADF) were determined with the approach employed by Van Soest et al. [24]. Calcium (Ca) and phosphorus (P) were analyzed using coupled plasma optical emissions spectrometry [23]. Following the operating methods of the oxygen bomb calorimeter (MTZW-4, Shanghai Mitong Electromechanical Technology Co., Ltd., Shanghai, China), the gross energy of the diet was measured.

### 2.6. Calculation

Following the prediction model of Moss et al. [25], ruminal CH_4_ production was estimated stoichiometrically, as shown in Equation (1):

CH_4e_ (mmol/L) = 0.45 × acetate − 0.275 × propionate + 0.40 × butyrate, (1)

CH_4_ production (L/d) was calculated as follows based on the prediction model of Patra et al. [26] via the gross energy intake (GEI, MJ/d):

GEI (MJ/d) = DMI × GE,(2)

CH_4_ (MJ/d) = 0.208 + 0.049 × GEI, (3)

CH_4_ (L/d) = 0.714 × CH_4_ (MJ/d)/0.05565, (4)

According to Equation (2), the GEI was calculated as a result. In addition, the conversion factor (55.65 kJ per g of CH_4_) was employed when Equation (3) used MJ/d. Using the molar density of CH_4_ (0.714 g/L), the g/d was converted to L/d. Following Equation (4), MJ/d was converted to L/d, which was expressed in the present study as CH_4_ production. Finally, the measurements of CH_4_ production (L/kg ADG) were calculated based on the ADG.

### 2.7. Statistical Analysis

Data related to growth performance, serum parameters, rumen fermentation, and bacterial alpha diversity were subjected to analysis with the generalized linear model (GLM) procedure of SAS (SAS Inst. Inc., Cary, NC, USA; version 9.4). The least-square means were compared with a multiple comparison test (Tukey/Kramer), and significance was declared at *p* < 0.05 in the linear and quadratic models. The correlations between the bacterial community at the genus level and rumen fermentation, blood serum index, and growth performance were determined using Spearman’s correlation analysis.

## 3. Results

### 3.1. Effect of Increasing CS Addition on the Growth Performance in Feedlot Lambs

**Figure 1:**
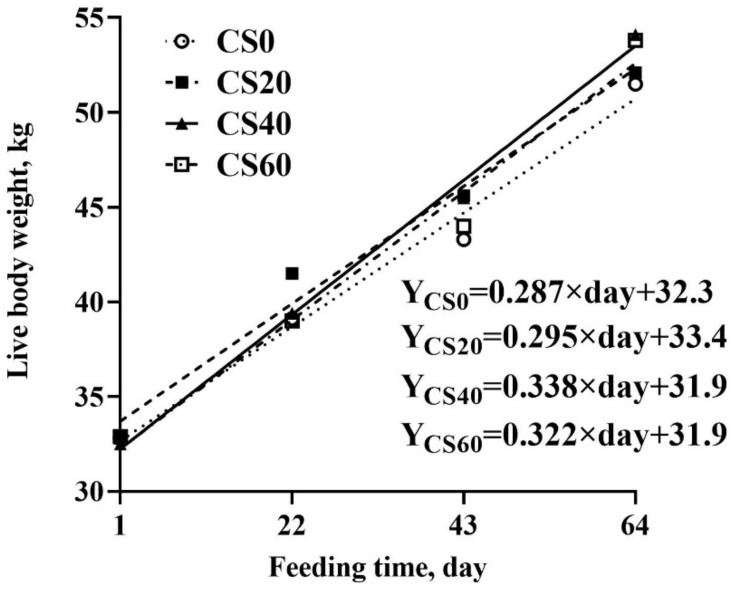
Effect of dietary CS addition on live body weight of feedlot lambs (CS0, control group; CS20, 20 mg/kg BW; CS40, 40 mg/kg BW, CS60, 60 mg/kg BW).

As shown in Table 2, increasing the CS addition linearly increased the ADG and quadratically increased the DMI (*p* < 0.05), and the greatest ADG and DMI values occurred at 40 mg/kg BW. Compared to CS0, increasing the CS addition increased the growth rate in lambs, and the highest growth rate was at 40 mg/kg BW (Figure 1). The ADG and DMI increased by 22.1% and 12%, respectively, compared to CS0 in the group fed 40 mg/kg BW. As a result, increasing the CS addition linearly decreased F:G (*p* < 0.01). However, the lowest value of F:G was observed in the group at 60 mg/kg BW.

### 3.2. Effect of Increasing CS Addition on Blood Serum Concentrations along the Growth Hormone Axis

As shown in Table 3, increasing CS addition linearly decreased the serum concentrations of somatostatin and leptin (*p* < 0.01), but it linearly increased the levels of GH and IGF-I (*p* < 0.01).

### 3.3. Effect of Increasing CS Addition on Rumen Fermentation Characteristics and Methane Production in Feedlot Lambs

As shown in Table 4, although increasing the CS addition did not alter the pH or ammonia N, the increase in CS addition linearly increased the MCP in the rumen (*p* < 0.01). The total VFA level increased linearly against the increase in CS addition (*p* < 0.01). Regarding the VFA in the molar proportion, increasing the CS addition greater than 20 mg/kg BW decreased the acetate production and linearly increased the propionate production (*p* < 0.05). As a result, the acetate-to-propionate ratio decreased linearly against the increase in the addition of CS (*p* < 0.01).

The estimated methane production (CH_4e_) quadratically decreased against the increase in CS (*p* < 0.05). Although the methane production calculated from the gross energy intake increased linearly (*p* < 0.01), the methane production per kg of ADG decreased quadratically against the increase in CS (*p* < 0.01). This was consistent with the CH_4e_ calculated from the VFA concentrations in the rumen.

### 3.4. Effect of Bacterial Diversity and Community in Response to the CS Addition

As shown in Table 5, a Good’s coverage value greater than 0.99 indicates that the ruminal liquid samples provided sufficient OTU coverage for the bacteria community analysis. No differences were observed in the Chao and Ace, which evaluate the total number of species and richness in samples. Moreover, increasing the CS supplementation did not significantly change the Shannon and Simpson index.

**Figure 2:**
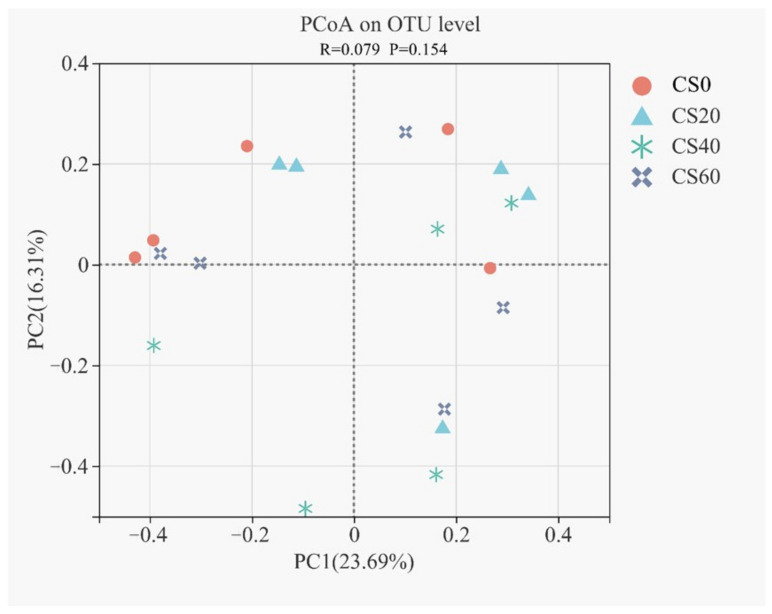
Principal coordinates analysis (PCoA) plot based on the Bray–Curtis dissimilarity matrix to compare the bacterial structure among feedlot lamb feed rations with different supplementary concentrations of cysteamine (CS0, control group; CS20, 20 mg/kg BW; CS40, 40 mg/kg BW; CS60, 60 mg/kg BW).

Based on the Bray–Curtis distance matrix, the beta diversity of the bacterial communities was evaluated via the principal coordinates analysis (PCoA, Figure 2). According to the overlapped clustering of bacteria in four groups, no clear distinction was observed from the PCoA analysis. Moreover, ANOSIM also exhibited some changes (R = 0.079, *p* = 0.154), suggesting that there was no significant effect on the structure of the bacterial communities.

As shown in Table 6, 21 bacterial phyla at the phylum level were annotated in the present study. Furthermore, *Firmicutes*, *Bacteroidota*, *Acitinobacteriota*, *Patescibacteria*, *Proteobacteria*, *Synergistota*, and *Spirochaetota* were determined to be the predominant phyla with a relative abundance of > 1%. Increasing the dietary CS addition did not affect the predominant phyla except for the quadratic increase in *Bacteroita* and the linear decrease in *Actinobacteriota* (*p* < 0.05). At the genus level, 282 genera were annotated in the present study, from which the 12 genera with relative abundances of > 5% were selected for statistical analysis, including *Norank_f_Eubacterium_coprostanoligenes_group*, *Prevotella*, *Olsenella*, *NK4A214_group*, *Norank_f_F082*, *Lachnospiraceae_NK3A20_group*, *Christensenellaceae_R-7_group*, *Acetitomaculum*, *Ruminococcus*, and *Rikenellaceae_RC9_gut_group*. Among these predominant genera, the relative abundance of *Prevotella* increased quadratically with the increase in CS addition, while the *Olsenella* and *Lachnospiraceae_NK3A20_group* decreased quadratically (*p* < 0.05). Additionally, no significant difference was observed in the other genera among the four treatments.

### 3.5. Correlations among the Top 10 Bacterial Genera and the Parameters of Rumen Fermentation, Blood Serum Index, and Growth Performance

**Figure 3:**
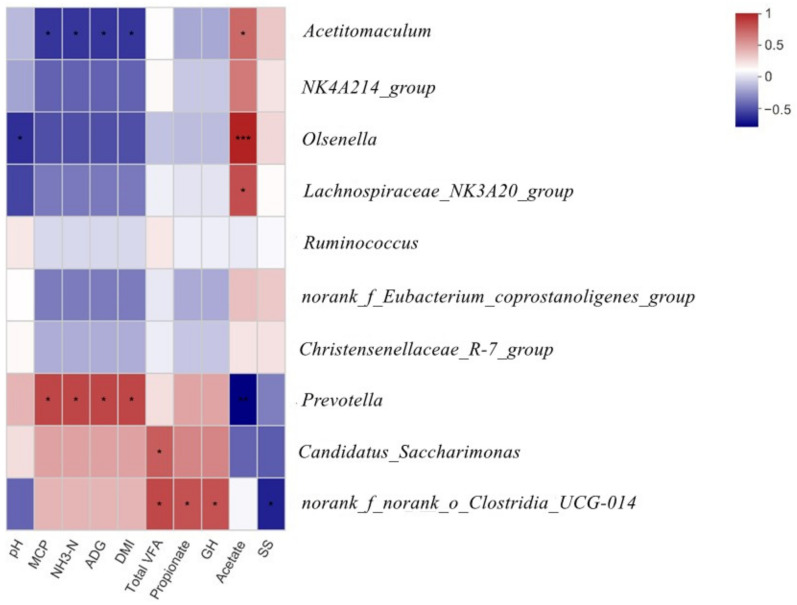
Heat map of the correlations among the top 10 bacterial genera and the parameters of rumen fermentation, blood serum index, and growth performance. Color intensity represents *p*-values of correlation, * *p* ≤ 0.05, *** *p* ≤ 0.01.

According to the heat map analysis shown in Figure 3, *Olsenella* was negatively correlated with the pH (r = −0.5, *p* < 0.05). The MCP, ammonia N, ADG, and DMI were negatively correlated with the presence of *Acetitomaculum* (r < −0.57, *p* < 0.05) but positively correlated with *Prevotella* (r > 0.68, *p* < 0.05). The total VFA, propionate, and GH were positively correlated with *norank_f_Eubacterium_coprostanoligenes_group* (r > 0.64, *p* < 0.05). In addition, *Candidatus_Saccharimonas* also presented a positive correlation with the total VFA (r = 0.60, *p* < 0.05). A positive correlation was observed between acetate and *Acetitomaculum*, *Olsenella*, and *Lachnospiraceae_NK3A20_group* (r > 0.55, *p* < 0.05), but it was negatively correlated with *Prevotella* (r = −0.74, *p* < 0.01). Furthermore, somatostatin concentrations in serum were negatively correlated with *norank_f__norank_o__Clostridia_UCG-014* (r = −0.64, *p* < 0.05).

## 4. Discussion

### 4.1. Growth Performance in Response to Dietary CS Addition and Its Possible Action Mechanism

In feeding practice, daily feed intake, BW gain, and feed conversation efficiency are vital parameters for determining whether a feed additive can improve growth performance. In accordance with the results obtained in previous studies, which showed that CS acted as a growth promoter in pigs, sheep, and beef cattle in terms of the DMI, ADG, and apparent nutrient digestibility [11,12,27], increasing the CS addition in the present study indeed exhibited a positive effect on growth performance. Du et al. [28] noted that the exogenous addition of CS promoted the secretion of ghrelin mRNA, which was considered to promote animal appetite and be an effective stimulant of gastric movement and gastric acid secretion [29]. However, in the present study, the increase in appetite could be attributed to the decrease in leptin concentrations. Increasing the dietary CS addition increased the ADG and decreased the F:G, suggesting that CS increased the growth rate and improved feed efficiency. A previous study demonstrated that 80 mg/kg of dietary additions daily or every third day significantly increased the BW gain and improved the feed conversion ratio in sheep, which was similar to the findings of our study [30].

Somatostatin is a peptide secreted by the hypothalamus that inhibits the GH secretion of GH from somatotrophs [31]. In the present study, increasing the CS addition decreased the concentration of somatostatin, suggesting the inhibitory effect of CS on somatostatin. Specifically, CS, with the reducibility of the thiol group [32], breaks the disulfide bond in the molecular structure of somatostatin. GH, a hormone secreted by the anterior pituitary, is the primary regulator of somatic growth via the secretion of IGF-I by the liver [33]. In the present study, the concentrations of GH and IGF-1 were improved by inhibiting somatostatin to promote animal growth. Compared to some rumen modifiers [34] and hormonal growth promoters [35], the present study indicates that CS could provide a new channel for promoting feed conversion efficiency by regulating the growth hormone-releasing hormone (HPGH) axis.

Leptin is a hormone secreted by adipose tissue [36] that acts on receptors in the central nervous system and participates in the promotion of foraging behavior and energy metabolism [37,38]. The decrease in leptin in response to dietary CS in the present study could explain why the feed intake was increased, as previously reported in a case of leptin being found to reduce the appetite and feed intake of animals through the central neural pathway [39]. Based on the feed trial results in the present study, a dietary CS addition of no less than 40 mg/kg BW is recommended for lamb feedlotting in feeding practice.

### 4.2. Rumen Fermentation Characteristics

An optimal ruminal pH is essential for maintaining a stable ruminal environment, and the appropriate pH for rumen is between 6.2 and 7.0 [40]. In the present study, the increasing addition of CS did not alter the rumen pH, which was within the normal range. Ammonia N is a vital intermediate of protein metabolism and a nitrogen source for MCP synthesis. The content of MCP increased linearly with the increasing dietary CS level, which was consistent with the numeric increase in ammonia N. VFAs are the main energy source of ruminants, providing 75% of their digestible energy and playing a pivotal role in various metabolisms [41]. In the present study, increasing the dietary CS addition linearly increased the total VFA concentration, implicating that CS indeed enhanced the rumen fermentation efficiency. An earlier study on a weaned lamb with fistulated rumen noted that dietary CS addition at a 150 mg/kg BW daily level resulted in a significant elevation of lactate dehydrogenase activity and total VFA [42], and the increase in the dehydrogenase activity indirectly implied the enhanced metabolic activity of the rumen microorganism. Moreover, an assay in vitro showed that rumen fermentation fluid incubated with CS supported higher VFA production than that of the control group [43]. The aforementioned evidence confirmed that dietary CS addition exhibited a positive effect on rumen fermentation, which should have explained the improvement in growth performance observed in the present study. As one of the fermentation products in the rumen, it is well known that propionate is the primary precursor of gluconeogenesis in ruminants, which makes a significant net contribution to their glucose synthesis [41]. CS was reported to participate in the increase in propionate in the rumen fermentation in vitro of goats, especially at 50 mg/L of CS addition [43]. The above results show that increasing dietary CS shifted the rumen fermentation pattern toward glucogenic propionate production, which could mean enhanced feed energy utilization efficiency.

As a potent kind of greenhouse gas, methane (CH_4_) is a significant contributor to global warming, and its production leads to energy loss in host animals of up to 12% [44,45]. Among animals, ruminants are the primary emitters of CH_4_ [46]. It has been reported that the formation of propionate consumed H_2_, while the production of acetate was related to the production of H_2_ [25]. In the present study, promoting the effect of propionate by adding CS supported a reduction in methane production. According to the results obtained in previous studies, when Merino × Dorset lambs were fed a diet adding CS at 80 mg/kg BW daily or every third day, the methane yield was significantly reduced [12]. In cattle, the methane yield was found to be significantly mitigated by supplying CS at 80 mg/kg BW daily in the diet [27]. In addition, some studies have reported that CS reduced the number of methanogens by inhibiting the number of protozoa [47,48], which might explain the inhibition of methane production. However, we observed an increase in methane production with increased CS based on L/day, which might be closely related to the increase in the DMI [49].

### 4.3. Bacterial Diversity and Community in Response to CS Addition

Species richness and the evenness of a sample or individual community are elaborated by alpha diversity [50]. In the present study, no difference emerged in the alpha diversity with an increasing supplementation of dietary CS. Furthermore, the pH was not affected by the supplementation of CS among all treatments. Thus, the stable diversity of rumen microbes could be explained by the stable pH, which directly affects the development of microorganisms [51].

At the phylum level, *Firmicutes*, *Bacteroidota,* and *Actinobacteriota* were the dominant phyla, which is consistent with Chen et al.’s findings [52]. At the genus level, *Prevotella* increased quadratically, while *Olsenella* and *Lachnospiraceae_NK3A20_group* decreased quadratically with the increase in CS supplementation. As the core genus of bacteria in the rumen, *Prevotella* plays a crucial role in digesting proteins, peptides, starch, etc. [53], which could lead to the secretion of enzymes that degrade starch and produce propionate or propionate-precursor succinate [54], thereby restricting fiber fermentation [55]. Moreover, *Prevotella* is an essential microbial member that participates in the degradation of peptides into amino acids, which is regarded as the limiting process in proteolysis [56,57] and may explain the increase in growth performance (e.g., body weight gain) in our study. A high abundance of *Prevotella* rapidly and efficiently degrades soluble carbohydrates into glucose, and then, through the glycolysis pathway, it produces pyruvate, which is the main precursor for VFA production [58,59]. In the present study, the promoting effect of CS on *Prevotella* was consistent with the higher molar ratio of propionate, which presented a positive correlation between *Prevotella* and the production of propionate [60]. Previous studies have shown that *Prevotella* increases glycogen storage [61], and propionate involved in the gluconeogenesis pathway provides more energy for the host [62]; together, these behaviors could explain the promoting effect of CS on growth performance. However, the addition of CS to the basal diet between growth-retarded yaks and growth-normal yaks had no significant effect on *Prevotella* [63], which could be attributed to the breed and diets of the host. In the present study, the relative abundance of *Olsenella* in the CS-free control group was higher than in previous studies [64,65], which might be due to the dietary supplementation of foxtail millet silage as roughage with the growth of lactate-producing bacteria, such as *Olsenella,* during ensiling. Compared to the control group, increasing the dietary supplementation with CS decreased *Olsenella*, suggesting the inhibitory effect of the production of lactate via inhibiting the development of *Olsenella*. As an intermediate, lactate participation in the pathway of glucose metabolism and lactate conversion to volatile fatty acids has been demonstrated [66]. Moreover, the exact pathway to the inhibition of *Olsenella* with increasing dietary CS needs further investigation.

### 4.4. Association among Growth Performance, Serum Hormones, and Change in Bacterial Composition

According to the correlation analysis, the pH values were negatively correlated with the presence of *Olsenella*, reflecting the tolerance of *Olsenella* to the low pH. The MCP, ammonia N, ADG, and DMI showed a positive correlation with *Prevotella* but a negative correlation with *Acetitomaculum*, implicating that *Prevotella* rather than *Acetitomaculum* exhibited a positive feeding effect for feedlot lambs. An earlier study is consistent with ours in that a significant increase in *Prevotella* was found to be positively correlated with voluntary feed intake and growth performance in a probiotic feeding experiment of neonatal calves [67]. It could be interpreted that propionic acid produced by *Prevotella* presented the highest energy utilization efficiency, leading to improved growth performance as a key substrate in the sugar isomerization pathway, followed by acetate and butyrate. In addition, the same trend was observed in the positive relation between *norank_f__norank_o__Clostridia_UCG-014* and parameters such as total VFA, propionate, and GH, suggesting that they have a beneficial effect on feed energy utilization efficiency. Furthermore, *Acetitomaculum*, *Lachnospiraceae_NK3A20_group*, and *Olsenella* [68] were positively related to acetate production in the present study.

## 5. Conclusions

In summary, dietary supplementation of cysteamine at less than 60 mg/kg BW increased the growth rate and improved the feed efficiency to varying degrees in feedlot lambs, and not less than 40 mg/kg BW can be considered the optimal dosage for feedlotting. Furthermore, cysteamine exhibited inhibitory effects on somatostatin, promoting the levels of GH and IGF-1 to obtain positive growth performance. In rumen fermentation, cysteamine inhibited the estimated methane production and shifted the rumen toward glucogenic propionate fermentation via enriching *Prevotella*-producing propionate.

## Figures and Tables

**Table 1:** Feed ingredients and chemical composition of basal total mixed ration on a dry matter (DM) basis.

Item	Content
Ingredient, g/kg DM	
Foxtail millet silage	100
Corn stover	100
Corn meal	500
Soybean meal	170
Rapeseed meal	80
Vitamin and mineral premix ^1^	50
Chemical composition, g/kg DM	
Crude protein	153
Ether extract	33
Neutral detergent fiber	203
Acid detergent fiber	102
Calcium	9.1
Phosphorus	3.6
Gross energy, MJ/kg	15.8

^1^ The mineral–vitamin premix provided nutrients per kilogram of product: vitamin A, 150,000 IU; vitamin D3, 50,000 IU; vitamin E, 500 IU; vitamin B1, 200 IU; Fe as FeSO_4_•H_2_O, 1800 mg; Mn as MnO, 1500 mg; Zn as ZnO, 1000 mg; I as KI, 10.0 mg; Se as Na_2_SeO_3_, 3 mg; Co as CoSO_4_, 5 mg; Ca as Ca_2_CO_3_, 100 g; Phosphorus, 3 g; NaCl, 100 g.

**Table 2:** Effect of dietary cysteamine (CS) addition on growth performance and feed efficiency of feedlot lambs.

Item ^1^	Supplementary Level (mg/kg BW) ^2^				SEM	*p*-Value ^3^	
	CS0	CS20	CS40	CS60		Linear	Quadratic
ADG (g/d)	256 ^b^	276 ^ab^	313 ^a^	307 ^ab^	16.7	0.02	0.46
DMI (kg/d)	1.33 ^c^	1.34 ^c^	1.49 ^a^	1.38 ^b^	0.02	<0.01	<0.01
F:G	5.18 ^a^	4.83 ^b^	4.77 ^b^	4.48 ^c^	0.02	<0.01	0.12

^1^ ADG, average daily gain; DMI, dry matter intake; F:G, feed-to-gain ratio calculated as DMI divided by ADG. ^2^ Different supplementary level (CS0, control group; CS20, 20 mg/kg BW; CS40, 40 mg/kg BW, CS60, 60 mg/kg BW). ^3^ Linear and Quadratic represent linear and quadratic effects of CS addition. ^a–c^ Means within a row with different lowercase superscript letters differ at *p* < 0.05.

**Table 3:** Effect of dietary CS addition on serum hormones.

Item ^1^	Supplementary Level (mg/kg BW) ^2^				SEM	*p*-Value ^3^	
	CS0	CS20	CS40	CS60		Linear	Quadratic
Somatostatin	89.3 ^a^	82.7 ^b^	81.2 ^b^	76.9 ^b^	1.88	<0.01	0.53
GH	1.55 ^c^	1.99 ^b^	2.18 ^b^	2.44 ^a^	0.08	<0.01	0.29
IGF-1	36.6 ^c^	41.2 ^b^	42.1 ^b^	45.3 ^a^	0.93	<0.01	0.42
Leptin	2.97 ^a^	2.49 ^b^	2.42 ^b^	2.07 ^c^	0.08	<0.01	0.46

^1^ GH, growth hormone; IGF-1, insulin-like growth factor-1. ^2^ Different supplementary level (CS0, control group; CS20, 20 mg/kg BW; CS40, 40 mg/kg BW, CS60, 60 mg/kg BW). ^3^ Linear and quadratic represent linear and quadratic effects of CS addition. ^a–c^ Means within a row with different lowercase superscript letters differ at *p* < 0.05.

**Table 4:** Effect of dietary CS addition on rumen fermentation characteristics and methane production.

Item ^1^	Supplementary Level (mg/kg BW) ^2^				SEM	*p*-Value ^3^	
	CS0	CS20	CS40	CS60		Linear	Quadratic
pH	6.26	6.29	6.28	6.24	0.11	0.89	0.77
Ammonia N, mg/mL	22.8	25.0	26.8	26.4	2.43	0.24	0.59
MCP, mg/mL	0.377 ^b^	0.396 ^ab^	0.413 ^a^	0.405 ^a^	0.01	<0.01	0.07
Total VFA, mmol/L	118.3 ^b^	129.9 ^ab^	127.5 ^ab^	136.8^a^	4.16	0.01	0.79
VFA patterns, % molar							
Acetate	50.2 ^a^	47.8 ^ab^	46.8 ^b^	47.8 ^ab^	2.76	0.03	0.04
Propionate	22.9 ^c^	24.3 ^b^	25.6 ^a^	25.7 ^a^	2.40	<0.01	0.12
Butyrate	6.52	7.63	5.51	6.63	1.30	0.23	0.98
Acetate-to-Propionate	2.15 ^a^	1.98 ^ab^	1.83 ^b^	1.86 ^b^	0.06	<0.01	0.15
CH_4e_, mmol/L	19.1 ^a^	17.6 ^b^	16.2 ^c^	16.7 ^bc^	0.43	<0.01	0.04
Methane, L/day	15.8 ^b^	15.9 ^b^	17.5 ^a^	17.5 ^a^	1.62	<0.01	0.69
Methane, L/kg ADG	61.9 ^a^	57.7 ^b^	55.9 ^c^	57.1 ^b^	0.23	<0.01	<0.01

^1^ VFA, volatile fatty acids; MCP, microbial crude protein; CH_4e_, estimated methane production. ^2^ Different supplementary level (CS0, control group; CS20, 20 mg/kg BW; CS40, 40 mg/kg BW, CS60, 60 mg/kg BW). ^3^ Linear and Quadratic represent linear and quadratic effects of CS addition. ^a–c^ Means within a row with different lowercase superscript letters differ at *p* < 0.05.

**Table 5:** Effect of dietary CS addition on ruminal bacteria alpha diversity based on OTUs.

Item	Supplementary Level (mg/kg BW) ^1^				SEM	*p*-Value ^2^	
	CS0	CS20	CS40	CS60		Linear	Quadratic
Coverage	0.99	0.99	0.99	0.99	<0.01	0.77	0.02
Chao	446	401	444	504	46.65	0.34	0.29
Ace	461	396	445	508	45.99	0.37	0.17
Shannon	3.47	3.25	3.37	3.84	0.21	0.21	0.13
Simpson	0.07	0.09	0.11	0.05	0.01	0.64	0.08

^1^ Different supplementary level (CS0, control group; CS20, 20 mg/kg BW; CS40, 40 mg/kg BW, CS60, 60 mg/kg BW). ^2^ Linear and Quadratic represent linear and quadratic effects of CS addition.

**Table 6:** Microbial community analysis at the phylum level (relative abundance > 1%) and genus level (relative abundance > 5%) of microbiomes obtained from rumen fluids of lambs.

Item	Supplementary Level (mg/kg BW) ^1^				SEM	*p*-Value ^2^	
	CS0	CS20	CS40	CS60		Linear	Quadratic
Phylum, %							
*Firmicutes*	58.1 ^ab^	68.5 ^a^	28.3 ^b^	63.7 ^ab^	11.4	0.64	0.28
*Bacteroidota*	4.08 ^a^	14.37 ^b^	60.92 ^b^	19.25 ^b^	8.35	0.03	0.01
*Actinobacteriota*	36.42 ^a^	3.40 ^b^	1.36 ^b^	3.21 ^b^	7.17	<0.01	0.03
*Patescibacteria*	0.60	3.74	3.93	2.32	1.80	0.50	0.21
*Proteobacteria*	0.08	8.62	2.17	0.87	3.97	0.81	0.23
*Synergistota*	0.07	0.71	1.22	0.15	0.54	0.74	0.13
*Spirochaetota*	0.10	0.19	1.34	0.11	0.61	0.66	0.29
Genus, %							
*Norank_f_Eubacterium_coprostanoligenes_group*	14.19	19.23	5.33	9.38	5.62	0.26	0.93
*Prevotella*	1.54 ^b^	8.54 ^b^	44.58 ^a^	9.45 ^b^	9.57	0.17	0.04
*Olsenella*	33.81 ^a^	1.04 ^b^	0.76 ^b^	11.20 ^ab^	7.98	0.07	0.02
*Lachnospiraceae_NK3A20_group*	12.83	4.20	3.60	9.35	3.10	0.42	0.04
*Christensenellaceae_R-7_group*	6.92	12.05	4.71	2.89	5.75	0.44	0.55
*Acetitomaculum*	5.02	5.29	0.41	3.92	2.14	0.39	0.46
*Ruminococcus*	2.71	7.13	1.83	3.53	3.25	0.84	0.68
*Norank_f_F082*	0.52	3.09	2.35	2.04	1.23	0.48	0.26
*NK4A214_group*	3.15	3.04	0.64	4.58	2.02	0.83	0.33
*Candidatus_Saccharimonas*	0.60	3.74	3.92	1.85	1.78	0.61	0.17
*norank_f__norank_o__Clostridia_UCG-014*	0.62	1.31	1.20	4.18	1.26	0.08	0.37
*Rikenellaceae_RC9_gut_group*	1.44	0.56	2.74	0.72	1.31	0.99	0.66

^1^ Different supplementary level (CS0, control group; CS20, 20 mg/kg BW; CS40, 40 mg/kg BW, CS60, 60 mg/kg BW). ^2^ Linear and Quadratic represent linear and quadratic effects of cysteamine addition. ^a,b^ Means within a row with different lowercase superscript letters differ at *p* < 0.05.

## Data Availability

Not applicable.
